# The diversity of cultivable endophytic fungi of the sand coast plant *Ipomoeapes-caprae* in Taiwan

**DOI:** 10.3897/BDJ.11.e98878

**Published:** 2023-02-17

**Authors:** Yu-Hung Yeh, Roland Kirschner

**Affiliations:** 1 National Taiwan University, Taipei, Taiwan National Taiwan University Taipei Taiwan

**Keywords:** biodiversity, coast plant, endophytic fungi, sandy beach

## Abstract

**Background:**

*Ipomoeapes-caprae* is a plant of sand coasts and it can tolerate stresses, such as high salinity, strong wind and sand movements and lack of nutrients. It plays an important role in coast protection and preventing erosion. Fungal endophytes show high biodiversity and have a strong influence on the survival of plants under different stress factors. Although this plant is important for sand coast ecosystems, little is known about the associated fungi. In this study, we isolated and identified endophytic fungi of *Ipomoeapes-caprae*, a dominant plant along the shore of Taiwan. The dataset contains 896 records, which correspond to 177 species. The geographical scope of the dataset covers the northern subtropical area of the main island of Taiwan, with its sand coasts in New Taipei, Taoyuan, Hsinchu and Taichung and two botanical gardens in Taipei and Taichung. The detailed original data of fungal diversity are rarely publicly shared under strictly formalised and, thus, reusable standards. As an example for such an approach, the complete occurrence dataset was made available in the Darwin Core Archive format via the Global Biodiversity Information Facility (GBIF) under Version 1.13, Taiwan Biodiversity Information Facility (TaiBIF) https://doi.org/10.15468/9h9rcg. In this first data paper on endophytic fungi, the scientific name and associated DNA sequence in the dataset were directly linked to other free online resource (Index Fungorum, GenBank), which shows the potential of GBIF for linking together different online data repositories.

**New information:**

We describe a dataset, in which the diversity of endophytic fungi of the sand coast plant *Ipomoeapes-caprae* in Taiwan was investigated.

## Introduction

Taiwan has abundant coastal terrains. The most widespread coastal ecosystems are sand dunes and sandy beaches especially along the northern and western coast of the main island. Herbaceous vegetation at sand coasts consists of specific plants which can tolerate high salinity, strong wind and sand movements and lack of nutrients and is, therefore, important for coast protection and preventing erosion (Chen et al. 2004). *Ipomoeapes-caprae* (L.) R.Br. (Convolvulaceae) is one of the most common and most widely distributed coastal plants in Taiwan (Chen et al. 2004). This species is an efficient sand binding plant forming large colonies and stabilising dunes (Devall 1992). Only a few records of endophytic fungi from *I.pes-caprae* have been published from India and Malaysia (Beena et al. 2000; Paliany et al. 2014). Most of these fungi isolated from this plant were tentatively identified to the genus level by sequences of the internal transcribed spacer sequences of the nuclear ribosomal RNA genes (ITS). Only a few isolates were identified to the species level.

Endophytes are microorganisms, mainly fungi, growing within plant tissues for all or part of their life cycle, but causing no apparent disease (Wilson 1995). According to the interactions between endophytic fungi and their host plants, endophytic fungi can be divided into three groups, mutualists, parasites and commensalists (Jia et al. 2016; Kirschner 2018). In mutualism, certain endophytic fungi can confer tolerances to abiotic and biotic stresses on their host plants, enhance their growth and suppress diseases (Redman et al. 2002; Rodriguez et al. 2008). For most endophytes, however, the roles they play for their host plants are not known. After the discovery that an endophytic fungus isolated from the bark of *Taxusbrevifolia* Nutt. was able to produce the anticancer compound taxol (Zhao et al. 2010), endophytic fungi have also become major potential sources in the search for bioactive compounds and drug development (Jia et al. 2016). In traditional biological conservation, we focus on the plant, not the microorganisms associated with this plant from a special habitat. When the habitat is destroyed, the plant can be preserved ex situ in botanical gardens, but the fate of microbes associated with this plant has not been investigated.

The aim of the study was to study cultivable endophytic fungi of *I.pes-caprae* and to analyse whether fungi were specific to plant species and plant organs (root, stem, leaf). We also collected *I.pes-caprae* from botanical gardens located in the inland of the island for comparing the fungal communities between natural and artificial habitats in order to obtain insights about the influence of the habitat on the occurrence of endophytic fungi. The study of composition and diversity of fungi from *I.pes-caprae* provided new data about the geographical and host distribution of fungi. New isolates and DNA data were deposited for future systematic and biotechnological studies and applications.

Although studies of endophytic fungi yield high numbers of strains, species and associated DNA sequence data, the complete original data are often not presented in detail in publications; or when provided (usually as supplementary materials), the standards of species identification, as well as selection and format of data, vary between different publications. The divergent standards limit the reusability of the data. On the other hand, the data of a single mycological diversity study become scattered in different repositories, such as strain numbers (strain catalogues), specimens numbers (collection databases), DNA sequence accession numbers (DNA databases) and repositories of scientific names. The Darwin Core within the Global Biodiversity Information Facility (GBIF) allows bringing back such data into a single dataset in a unified format so that datasets from different researchers, areas and times can be analysed together (Paton et al. 2020). The number of digitised fungal collections with searchable online databases has been increasing, but only 21% plant and fungal collections are available through GBIF; fungal specimens are much less represented than plants; and tropical Asia is amongst the under-represented regions (Paton et al. 2020). The aim of this data paper, therefore, is to present our original data in the strictly standardised format of the Darwin Core within GBIF as an example for preserving and sharing the original data of endophytic fungi. The hitherto single dataset about endophytic fungi deals with fungi from trees in Norway (Norden and Andreasen 2022). The potential of GBIF to enhance interoperability between the different resources (Osawa 2019) was particularly considered by our resource selection. Our complete occurrence dataset (Yeh and Kirschner 2022) was made available in the Darwin Core Archive format via the Global Biodiversity Information Facility (GBIF) under Version 1.12. Taiwan Biodiversity Information Facility (TaiBIF) https://doi.org/10.15468/9h9rcg.

**Potential for reuse and interoperability of the dataset**

As far as we know, complex original data of endophytic fungi have hitherto not been published in the form of a data paper, based on a GBIF dataset. GBIF is a system that helps to mobilise digitised data globally for biodiversity informatics (Paton et al. 2020). The Darwin Cores of GBIF provide unified standards, which are crucial for reuse and interoperability when different datasets are connected (Osawa 2019). By enhanced reusability, single-time data or data from different places can be connected, which allows ecological comparison beyond time and space of the individual study (Osawa 2019). The geographical coverage of our study was limited to northwest Taiwan, while the host plant has pantropical distribution (Devall 1992). The anecdotal records of endophytes from *I.pes-caprae* in India and Malaysia (Beena et al. 2000; Paliany et al. 2014) indicate the potential for geographical extension.

Primary biodiversity data can principally be divided into “specimen-based” data with high cost and high quality and the low cost and low quality “observation” (Osawa 2019). Due to the relatively difficult species identification, fungal species identification can hardly be obtained by field observation alone. Gathering fungal biodiversity data tend to be highly cost- and time-intensive, particularly when cultivation and DNA sequencing for more than one barcode region are involved. A good compromise between high accuracy by cost-intensive molecular identification or microscopical morphological determination and limited funding is choosing representative strains of morphospecies for DNA sequencing and treating the other presumably conspecific strains under “observation”. The representative strains are deposited as “specimens” in a living collection and/or as dried specimens in a museum. We feel that the present Darwin Cores do not yet resolve the optimal distinction of separate “PreservedSpecimen”, “LivingSpecimen” and combinations of both. In the latter case, we chose “otherCatalogNumbers” for the living strains in addition to the preserved museum specimens. This combination accounted for the majority of our strains, while, in only five living strains, no museum specimen was deposited. In terms of data quality, in addition to using barcodes other than the ITS region for enhancing the resolution of species identification, the control of surface disinfection is crucial for verifying the endophytic nature of the fungi. The only dataset about endophytic fungi in GBIF deals with fungi from trees in Norway (Norden and Andreasen 2022). The fungi were investigated by a combined approach of field collection, observation, cultivation and barcoding and metabarcoding approach, based on the ITS region. The taxonomic entries in the dataset were mainly only linked to geodetic and time data and sample numbers, without information about the host tree species, origin of sample (whether collected on bark or derived from wood bore core) and DNA sequences (Norden and Andreasen 2022). Due to the low reproducibility of the metabarcoding, as well as high proportion of human observation, lack of additional barcodes and missing data entry, the reusability of this dataset is quite limited.

Due to the inflation of taxonomic names and rapid changes of names, we consider the Darwin Core “scientificNameID” to be particularly important, because it allows a direct link of a scientific name to a constantly updated name repository. For enhancing interoperability of our dataset, scientific names and DNA sequences were directly linked to the databases Index Fungorum and GenBank so that updates in these databases can be retrieved conveniently. Although for specimens and strains, accession numbers are available in certain online collection databases, there was no option for linking them directly. Many not yet fully identifiable or presumably conspecific strains and specimens could not be deposited in the collections according to their policies due to limited capacities. These deficits of professional collections indicate the potential for their future improvement by adequate funding and management. Vouchers that could not be deposited in the future may be found to represent cryptic species, which will hardly be resolved under the present policies.

The English spelling of place names is very confusing, particularly in Taiwan, where names changed during the Japanese rule and after the retrocession, while currently different spellings also exist for the same place. For pragmatic reasons, we chose the spelling used in the online database of the national postal service. Using the same place names as the local postal service may also be useful in other countries without widely accepted unified English spelling of names.

## Project description

### Title

1. The effect of ex-situ preservation on the diversity of endophytic fungi in the coastal plant *Ipomoeapes-caprae*.

2. Taxonomic study of fungi on marattioid ferns in Taiwan I & II.

### Personnel

Yu-Hung Yeh - School of Forestry & Resource Conservation, National Taiwan University, 10617 Taipei City, Daan District, Roosevelt Rd. Sec. 4 No. 1.

### Funding

1. Ministry of Science & Technology of Taiwan (MOST 108-2621-B-002-007).

2. Ministry of Science & Technology of Taiwan (MOST 109-2621-B-002-004, 110-2621-B-002-001-MY2).

## Sampling methods

### Sampling description

One individual plant was collected per sampling (twice per year, representing the hot summer in one sampling event and another, cooler season from autumn to spring for the other collection event). Plants which were not conspicuously buried by sand during the collection time were removed with a trowel, individually placed in bags, returned to the laboratory and kept at 4°C until further processing for endophyte isolation within 48 hours after sampling. Altogether, 37 individuals of *I.pes-caprae* were collected from eight sites (27 from New Taipei, Taoyuan, Hsinchu and Taichung; 10 from Taipei and Taichung Botanical Gardens). Freshly collected healthy plants were cut into fragments (leaves, stems, roots) and surface-sterilised. Plant fragments were surface-sterilised under sterile conditions by agitation in 95% ethanol for 1 minute, 6–12% sodium hypochlorite (Hung Ei Chemical Co., Ltd., Taipei) for 3 minutes (for stems) or 1.5 minutes (for roots and leaves), 95% ethanol for 0.5 minutes and then rinsed in sterile water. All stems and roots were cut into six segments of approximately 1–2 cm and each leaf into the petiole, as well as three segments of ca. 0.6 cm diam. from the lamina. Three segments of stems and roots and two segments of the leaf lamina and the petiole were immediately placed on to malt extract agar (MEA) or corn meal agar (CMA) with 0.2% chloramphenicol. All isolates obtained from each plant sample were classified according to their morphological appearance into morphotypes. Representative isolates were identified to species as far as possible and deposited in the Bioresource Collection and Research Center, Hsinchu, Taiwan (BCRC). Dried cultures were deposited in the fungal specimen collection of the National Museum of Natural Science, Taichung, Taiwan (TNM). Since BCRC and TNM presently do not accept several specimens or strains from the same species, nor do they deposit collections without full scientific names of species or genus, samples which did not meet the criteria for deposit were not noted as PreservedSpecimen, but only as HumanObservation in the column basisOfRecord.

### Quality control

Since surface-sterilisation has to be adjusted whenever endophytes are isolated from a plant species which was hitherto uninvestigated with respect to endophytes, we optimised the methods for surface sterilisation for the different plant parts of *I.pes-caprae*. The effectiveness was further controlled regularly by the imprint technique, i.e. by pressing the surface-sterilised plant fragments on to control media after surface disinfection, before placing the fragments on to other media for growth of the fungi. Only if no growth occurred on the control media, but in the media with plant fragments, then the surface sterilisation procedure was neither too weak nor too rigorous (Rodriguez et al. 2008). The composition of media may influence the isolation frequency endophytic fungi. Some hyphal fungi grow out vigorously from the same plant fragment and too quickly overgrow slowly growing other hyphal fungi and yeasts. If hyphal fungi are common endophytes in a given plant (as in our study), a rather nutrient-poor medium is preferable because then the hyphal fungi grow more slowly than in nutrient-rich media. Loss of slowly-growing hyphal and yeast-like fungi through suppression by too dominant fungi is reduced. We isolated about ten yeast species, such as in the basidiomycetous genera *Cryptococcus*, *Cystobasidium*, *Graphiola*, *Malassezia*, *Moesciomyces*, *Pseudozyma* and *Rhodosporidiobolus* and ascomycetous “black” yeasts in the genera *Aureobasidium* and *Hortaea*, as well as in the saccharomycetalean genus *Starmerella* so that quite diverse yeasts were detected with our method. DNA sequencing was performed by Mission Biotech (Nankang, Taipei) with the same primers used for the PCR. The forward and reverse DNA sequences were edited using CodonCode Aligner version 4.0.1 (CodonCode Corp., USA). Sequences with sufficient publication quality and length and were submitted to GenBank and the DNA Data Bank of Japan. The sequences were subjected to BLAST searches at GenBank (https://blast.ncbi.nlm.nih.gov/). Depending on the similarities of the used barcode and verifiability by morphology, the strains were identified to species, genus or higher taxonomic ranks. As mentioned before, barcodes other than rDNA sequences were applied when necessary in order to enhance the identification (Table 1). Only sequences cited in publications were considered for identification. Scientific names were crosschecked and updated with Index Fungorum (http://www.indexfungorum.org)

### Step description

Strains of endophytic fungi were identified by both morphological and molecular characteristics. The following characteristics were used in the characterisation and identification of morphospecies: colony appearance, colour and structure of mycelia and in anamorphic fungi the type of conidiogenous cells, conidiophores and conidia. The microscopic samples were mounted in 5–10% (w/v) aqueous potassium hydroxide (KOH) solution. For molecular identification of fungi, DNA sequences of the internal transcribed spacer regions (ITS) and/or the ribosomal large subunit RNA gene (LSU rDNA) were generated for a preliminary identification. Additionally, depending on the species resolution in certain genera, where rDNA barcodes are insufficient, other barcodes were applied. Genomic DNA was extracted with Genomic DNA Spin Kit (Bioman Scientific Co., Ltd., Taiwan) according to the manufacturer’s protocol. The PCR products were synthesised with different primers, based on published phylogenetic analyses of specific genera or species complexes of fungi (Table 1). The PCR mixture consisted of 12.5 μl 2x GoTaq Green Master Mix (Promega, Genelabs Life Science Corp., Taiwan), 1 μl of primer pairs, ddH_2_O (up to 25 μl) and 5 μl genomic DNA. The ITS/LSU sequence amplification conditions were 10 cycles of 94°C for 30 s, 60°C to 50°C (decreasing 1°C per cycle) for 30 s; 72°C for 1 min, followed by 28 cycles of 94°C for 30 s; 50°C for 30 s; 72°C for 1 min; and finally 72°C for 1 min. The TUB sequence amplification conditions were an initial temperature 94°C for 5 min, followed by 40 cycles of 94°C for 45 s, 52°C for 30 s and 72°C for 90 s, with final elongation of 6 min at 72°C. The HIS3 sequence amplification conditions were an initial temperature 93°C for 5 min, followed by 40 cycles of 93°C for 30 s, 53°C for 30 s and 72°C for 2 min. The amplification conditions for the fatty acid elongase 1 gene (ELO) were initial denaturation of 2 min at 94°C, followed by 6 cycles of 15 s at 94°C, 15 s at 58°C and of 45 s at 72°C and 30 cycles of 15 s at 94°C, 15 s at 56°C and of 45 s at 72°C, with an elongation of 7 min at 72°C. The TEF sequence amplification conditions were an initial temperature of 94°C for 5 min, followed by 40 cycles of 94°C for 45 s, 52°C for 30 s and 72°C for 90 s, with final elongation of 6 min at 72°C. The RPB2 sequence amplification conditions were an initial temperature of 95°C for 5 min, followed by 30 cycles of 95°C for 1 min, 50°C for 2 min and 72°C for 2 min, with final elongation of 10 min at 72°C. The amplified products were checked with electrophoresis in a 2% agarose gel subsequently stained with SafeView DNA Stain (GMbiolab Co., Ltd., Taiwan) and visualised under UV light (312 nm).

## Geographic coverage

### Description

*Ipomoeapes-caprae* was collected from the six beach sites and two botanical gardens in the northern and central parts of the main island of Taiwan characterised by a subtropical climate. The dataset covers natural vegetation at sand coasts of New Taipei City (Bali District and Shihmen District), Taoyuan City (Guanyin District), Hsinchu County (Xinfeng Township) and Taichung City (Daan District and North District) and artificial plantations in the botanical gardens of Taipei City and Taichung City. 1. Taipei Botanical Garden in Taipei City (Latitude: 25.032205 Longitude: 121.509884) 2. Taichung Botanical Garden in Taichung City (Latitude: 24.156172 Longitude: 120.666314) 3. Wazihwei Beach in New Taipei City (Latitude: 25.168699 Longitude: 121.414081) 4. Shimen Beach in New Taipei City (Latitude: 25.292212 Longitude: 121.544561) 5. Daan Beach in Taichung City (Latitude: 24.379000 Longitude: 120.583286) 6. Nanpu Beach in Taichung City (Latitude: 24.343201 Longitude: 120.560261) 7. Sandy beach at Fongkeng fishing port in Hsinchu County (Latitude: 24.90521 Longitude: 120.96632) 8. Guanyin Beach in Taoyuan City (Latitude: 25.047 Longitude: 121.076)

### Coordinates

24.156172 and 25.292212 Latitude; 120.583286 and 121.414081 Longitude.

## Taxonomic coverage

### Description

This dataset contains data from the Kingdom Fungi, Divisions Ascomycota, Basidiomycota and Zygomycota, corresponding to a total of 13 classes and 33 orders. It includes 177 different species of fungi for a total of 896 fungal strains. Classes and orders included in the dataset are given in Table 2.

## Temporal coverage

### Notes

Samples of *I.pes-caprae* were collected in Taiwan during the years 2013–2015 and 2019–2020.

## Usage licence

### Usage licence

Creative Commons Public Domain Waiver (CC-Zero)

## Data resources

### Data package title

The diversity of cultivable endophytic fungi of the sand coast plant *Ipomoeapes-caprae* in Taiwan.

### Resource link

https://www.gbif.org/dataset/42b98a7f-4252-42a0-a56e-069df3eb3a7d

### Number of data sets

1

### Data set 1.

#### Data set name

The diversity of endophytic fungi of the sand coast plant *Ipomoeapes-caprae* in Taiwan.

#### Data format

Darwin Core

#### Description

This dataset contains data from the Kingdom Fungi, Divisions Ascomycota, Basidiomycota and Zygomycota from Taiwan. It contains 896 records, which correspond to 177 species. The Darwin Core definitions are arranged according to their sequence in the dataset and Darwin Core Maintenance Group (2021) in some cases with some specific modifications.

**Data set 1.** 

Column label	Column description
OccurrenceID	An identifier for the Occurrence (the occurrenceID globally unique).
basisOfRecord	The nature of the related resource. Three kinds of basisOfRecord were used in this study. 1. PreservedSpecimen: A fungal specimen was preserved as living or dried culture. 2. LivingSpecimen: Only a living strain was preserved. 3. HumanObservation: Only human observation was based on morphospecies classification of a strain without preserving a permanent fungal specimen. Since the collections BCRC and TNM presently do not accept several specimens or strains from the same species, nor are collections without full scientific names of species or genus permitted, samples which did not meet the criteria for deposit or for other reasons could not be deposited (because of loss due to contamination etc.) were recorded as HumanObservation only.
scientificName	The full scientific name of isolates was given as in Index Fungorum (http://www.indexfungorum.org/).
scientificNameID	An identifier for the nomenclatural details of a scientific name. The full scientific name of isolates was directly linked to the Index Fungorum Registration Identifier (http://www.indexfungorum.org/).
identificationQualifier	A brief phrase or a standard term (e.g.: "sp. 1", "sp. 2", or “aff.”) to express the determiner's doubts about the scientific name. In this study, this qualifier is used to distinguish different species of the same genus, while it was not possible to provide a full binomial name.
taxonRank	The taxonomic rank of the most specific name in the scientificName. In this study, it expresses the taxonomic rank of the identification. In species labelled with “aff.” in the identificationQualifier, the full scientific name was given in the scientificNameID and “species” in taxonRank according to the requirement of the Darwin Core. Adding “aff.” to the species name means that the species similar to, but distinct from, the given species.
kingdom	The full scientific name of the kingdom in which the taxon is classified.
phylum	The full scientific name of the phylum or division in which the taxon is classified.
class	The full scientific name of the class in which the taxon is classified.
order	The full scientific name of the order in which the taxon is classified.
associatedTaxa	Host plant associated with the fungi.
degreeOfEstablishment	Referring to the host plant whether it is native or introduced from its native place (coast) to an inland place (botanical garden).
occurrenceRemarks	Comments or notes about the Occurrence. In this study, this qualifier refers to the specific plant tissues from which endophytes were isolated.
country	The name of the country in which the Location occurs.
countryCode	The standard code for the country in which the Location occurs.
county	The full, unabbreviated name of the next smaller administrative region than stateProvince (county, city etc.) in which the Location occurs.
locality	The specific name of the collection place. The spelling of the Chinese names follows Chunghua Post Co., Ltd. (https://www.post.gov.tw/post/internet/Postal/index.jsp?ID=207).
decimalLatitude	The geographic latitude (in decimal degrees, using the spatial reference system given in geodeticDatum) of the geographic centre of a Location.
decimalLongitude	The geographic longitude (in decimal degrees, using the spatial reference system given in geodeticDatum) of the geographic centre of a Location.
geodeticDatum	The ellipsoid, geodetic datum or spatial reference system (SRS) upon which the geographic coordinates given in decimalLatitude and decimalLongitude are based.
coordinateUncertaintyInMetres	The horizontal distance (in metres) from the given decimalLatitude and decimalLongitude describing the smallest circle containing the whole of the Location. In our dataset, we estimated 100 m for the coastal sites and 3 m for the plantations of *I.pes-caprae* in the botanical gardens.
eventDate	The date-time during which collection occurred in the field.
recordedBy	The primary collector or observer responsible for recording the original occurrence.
identifiedBy	A list of names of people who identified the fungus.
institutionCode	The name (or acronym) in use by the institution having custody of the object(s) or information referred to in the record. In this study, it followed Index Herbariorum sweetgum.nybg.org/science/ih/herbarium-list) using TNM for the National Museum of Natural Science where dried fungal specimens were deposited.
catalogNumber	An identifier (preferably unique) for the record within the dataset or collection. In this study, it means the identifier of TNM as provided in the online collection database (http://collection.nmns.edu.tw/scripts/fungi.dll).
otherCatalogNumbers	Alternate fully qualified catalogue numbers for the same Occurrence in the current dataset. In this study, it means an identifier of the Bioresource Collection and Research Center (BCRC) where cultures were deposited and can be retrieved under the identifier (https://catalog.bcrc.firdi.org.tw/) in addition to the PreservedSpecimen.
recordNumber	The personal identifier of representative isolates in this study.
associatedSequences	The URI of genetic sequence information associated with the Occurrence with direct link to GenBank (https://www.ncbi.nlm.nih.gov/nucleotide/).

## Figures and Tables

**Table 1.** Primers used in this study with the abbreviated names for the gene and forward and reverse primer, primer sequence and reference.

DNA region*	Primer name	Primer sequence (5’-3’)	References
ITS	ITS1F	CTTGGTCATTTAGAGGAAGTAA	(Gardes and Bruns 1993)
ITS	ITS4	TCCTCCGCTTATTGATATGC	(White et al. 1990)
ITS	ITS5	GGAAGTAAAAGTCGTAACAAGG	(White et al. 1990)
LSU	NL1	GCATATCAATAAGCGGAGGA AAAG	(Kurtzman and Robnett 1997)
LSU	NL4	GGTCCGTGTTTCAAGACGG	(Kurtzman and Robnett 1997)
TUB	TUB2Fd	GTBCACCTYCARACCGGYCAR TG	(Groenewald et al. 2013; Woudenberg et al. 2009)
TUB	TUB4Rd	CCRGAYTGRCCRAARACRAAGTTG TC	(Groenewald et al. 2013; Woudenberg et al. 2009)
TUB	Bt2a	GGTAACCAAATCGGTGCTGCTTTC	(Glass and Donaldson 1995)
TUB	Bt2b	ACCCTCAGTGTAGTGACCCTTGGC	(Glass and Donaldson 1995)
HIS3	H3F1	TGGCAAGGCCCCTCGCAAGC	(Bills et al. 2009)
HIS3	H3R1	TTGGACTGGATRGTAACACGC	(Bills et al. 2009)
ELO	ELO2-F	CACTCTTGACCGTCCCTTCGG	(Zalar et al. 2008)
ELO	ELO2-R	GCGGTGATGTACTTCTTCCACCAG	(Zalar et al. 2008)
RPB2	RPB2-F	GACGACCGTG ATCACTTTGG	(van den Brink et al. 2011)
RPB2	RPB2-R	CCCATGGCCTGTTTGCCCAT	(van den Brink et al. 2011)
TEF	EF1	ATGGGTAAGGARGACAAGAC	(O’Donnell et al. 1998)
TEF	EF2	GGARGTACCAGTSATCATGTT	(O’Donnell et al. 1998)
TEF	EF1-728F	CATCGAGAAGTTCGAGAAGG	(Carbone and Kohn 1999)
TEF	EF1-986R	TACTTGAAGGAACCCTTACC	(Carbone and Kohn 1999)
TEF	TEF-F	GCCCCCGGCCATCGTGACTTCAT	(van den Brink et al. 2011)
TEF	TEF-R	ATGACACCGACAGCGACGGTCTG	(van den Brink et al. 2011)

*Abbreviations of DNA regions/genes: ELO: Fatty acid elongase 1 gene, HIS3: Histone 3 gene, ITS: Internal transcribed spacer of the rRNA genes (partial SSU rRNA gene, ITS1, 5.8S rRNA gene, ITS2, partial LSU rRNA gene), LSU: Large-subunit rRNA gene, RPB2: RNA polymerase II second largest subunit genes, TEF: Translation elongation factor 1-α gene, TUB: β-tubulin gene.

**Table 2.** Classes and orders of fungi in this dataset.

Classes	Orders
Agaricomycetes	Agaricales, Cantharellales, Corticiales, Polyporales, Hymenochaetales
Agaricostilbomycetes	Agaricostilbales
Cystobasidiomycetes	Cystobasidiales
Dothideomycetes	Botryosphaeriales, Capnodiales, Dothideales, Mycosphaerellales, Pleosporales
Eurotiomycetes	Chaetothyriales, Eurotiales
Exobasidiomycetes	Exobasidiales
Leotiomycetes	Chaetomellales, Helotiales
Malasseziomycetes	Malasseziales
Microbotryomycetes	Sporidiobolales
Mucoromycetes	Mucorales
Saccharomycetes	Saccharomycetales
Sordariomycetes	Amphisphaeriales, Chaetosphaeriales, Diaporthales, Glomerellales, Hypocreales, Lulworthiales, Magnaporthales, Microascales, Sordariales, Xylariales
Tremellomycetes	Tremellales
Ustilaginomycetes	Ustilaginales

## References

[B8298918] Beena K. R., Ananda K, Sridhar K. R. (2000). Fungal endophytes of three sand dune plant species of west coast of India. Sydowia.

[B8295873] Bills Gerald F, Platas Gonzalo, Overy David P, Collado Javier, Fillola Asunción, Jiménez María Rosa, Martín Jesús, del Val Antonio González, Vicente Francisca, Tormo J Rubén, Peláez Fernando, Calati Kathleen, Harris Guy, Parish Craig, Xu Deming, Roemer Terry (2009). Discovery of the parnafungins, antifungal metabolites that inhibit mRNA polyadenylation, from the *Fusariumlarvarum* complex and other Hypocrealean fungi.. Mycologia.

[B8303236] Carbone I., Kohn L. M. (1999). A method for designing primer sets for speciation studies in filamentous ascomycetes. Mycologia.

[B8446155] Chen T. H., Yu H. M., Horng F. W. (2004). The movement of shifting sand and the growth of sand stabilizing plants at Shitsugan coast, Taoyuan. Quarterly Journal of Chinese Forestry.

[B8298935] Group Darwin Core Maintenance Simple Darwin Core. Biodiversity Information Standards (TDWG). https://dwc.tdwg.org/simple/.

[B8298943] Devall M. S. (1992). The biological flora of coastal dunes and wetlands. 2. *Ipomoeapes-caprae* (L.) Roth.. Journal of Coastal Research.

[B8295894] Gardes M., Bruns T. D. (1993). ITS primers with enhanced specificity for Basidiomycetes - application to the identification of mycorrhizae and rusts. Molecular Ecology.

[B8295912] Glass N L, Donaldson G C (1995). Development of primer sets designed for use with the PCR to amplify conserved genes from filamentous Ascomycetes. Applied and Environmental Microbiology.

[B8295930] Groenewald J Z, Nakashima C, Nishikawa J, Shin H-D, Park J-H, Jama A N, Groenewald M, Braun U, Crous P W (2013). Species concepts in *Cercospora*: spotting the weeds among the roses.. Studies in Mycology.

[B8295944] Jia Min, Chen Ling, Xin Hai-Liang, Zheng Cheng-Jian, Rahman Khalid, Han Ting, Qin Lu-Ping (2016). A friendly relationship between endophytic fungi and medicinal pants: A systematic review.. Frontiers in Microbiology.

[B8298952] Kirschner R. (2018). Fungi on the leaf – a contribution towards a review of phyllosphere microbiology from the mycological perspective.. Biochemical Systematics and Ecology.

[B8295956] Kurtzman C P, Robnett C J (1997). Identification of clinically important ascomycetous yeasts based on nucleotide divergence in the 5' end of the large-subunit (26S) ribosomal DNA gene. Journal of Clinical Microbiology.

[B8298969] Norden B., Andreasen M. Artsprosjekt Endofyttisk sopp i trær 23-19. v1.0. Norwegian Institute for Nature Research. Dataset/Occurrence. https://ipt.nina.no/resource?r=artsprosjekt_23_19_endophytes&v=1.0.

[B8295965] O’Donnell Kerry, Kistler H. Corby, Cigelnik Elizabeth, Ploetz Randy C. (1998). Multiple evolutionary origins of the fungus causing Panama disease of banana: Concordant evidence from nuclear and mitochondrial gene genealogies. Proceedings of the National Academy of Sciences.

[B8295974] Osawa Takeshi (2019). Perspectives on biodiversity informatics for ecology. Ecological Research.

[B8298977] Paliany A. S, Sivasothy Y., Awang K., Rizman-Idid M., Alias S. A. (2014). Marine derived fungi of Peninsular Malaysia – A biochemical perspective.. Chiang Mai Journal of Science.

[B8295983] Paton Alan, Antonelli Alexandre, Carine Mark, Forzza Rafaela Campostrini, Davies Nina, Demissew Sebsebe, Dröge Gabriele, Fulcher Tim, Grall Aurelie, Holstein Norbert, Jones Meirion, Liu Udayangani, Miller Joe, Moat Justin, Nicolson Nicky, Ryan Matthew, Sharrock Suzanne, Smith David, Thiers Barbara, Victor Janine, Wilkinson Tim, Dickie John (2020). Plant and fungal collections: Current status, future perspectives. Plants, People, Planet.

[B8298987] Redman R. S., Sheehan K. B., Stout R. G., Rodriguez R. J., Henson J. M. (2002). Thermotolerance conferred to plant host and fungal endophyte during mutualistic symbiosis.. Science.

[B8296010] Rodriguez Rusty J, Henson Joan, Van Volkenburgh Elizabeth, Hoy Marshal, Wright Leesa, Beckwith Fleur, Kim Yong-Ok, Redman Regina S (2008). Stress tolerance in plants via habitat-adapted symbiosis. The ISME Journal.

[B8296023] van den Brink Joost, Samson Robert A., Hagen Ferry, Boekhout Teun, de Vries Ronald P. (2011). Phylogeny of the industrial relevant, thermophilic genera *Myceliophthora* and *Corynascus*. Fungal Diversity.

[B8296042] White T. J., Bruns T., Lee S., Taylor J. (1990). Amplification and direct sequencing of fungal ribosomal RNA genes for phylogenetics. In: PCR - Protocols and Applications - A Laboratory Manual. USA, Academic Press..

[B8296051] Wilson Dennis (1995). Endophyte: The evolution of a term, and clarification of its use and definition. Oikos.

[B8296060] Woudenberg J. H.C., Aveskamp M. M., de Gruyter J., Spiers A. G., Crous P. W. (2009). Multiple *Didymella* teleomorphs are linked to the *Phomaclematidina* morphotype. Persoonia - Molecular Phylogeny and Evolution of Fungi.

[B8299006] Yeh Y. H., Kirschner R. The diversity of cultivable endophytic fungi of the sand coast plant *Ipomoea pes-caprae* in Taiwan. Version 1.12. Taiwan Biodiversity Information Facility (TaiBIF). Occurrence dataset.

[B8296070] Zalar P., Gostinčar C., de Hoog G. S., Uršič V., Sudhadham M., Gunde-Cimerman N. (2008). Redefinition of *Aureobasidiumpullulans* and its varieties. Studies in Mycology.

[B8299023] Zhao J., Zhou L., Wang J., Shan T., Zhong L., Liu X., Gao X., Mendez-Vilas A. (2010). Current research, technology and education topics in applied microbiology and microbial biotechnology.

